# Degradation of LiNi_0.5_Mn_1.5_O_4_ Cathodes in the P_111i4_FSI Ionic Liquid Electrolyte and Carbonate Electrolytes

**DOI:** 10.1021/acsami.5c11439

**Published:** 2025-09-08

**Authors:** Johan Hamonnet, Inger-Emma Nylund, Paraskevas Kontis, Weicheng Hua, Pedro Alonso-Sánchez, Juan Rubio Zuazo, Maria Valeria Blanco, Ann Mari Svensson

**Affiliations:** † Department of Materials Science and Engineering, 8018 Norwegian University of Science and Technology, NO-7491 Trondheim, Norway; ‡ Aragon Nanoscience and Materials Institute (CSIC-University of Zaragoza) and Department Condensed Matter Physics, Facultad de Ciencias, 50009 Zaragoza, Spain; § Materials Science Institute of Madrid (ICMM-CSIC), 3 Fuencarral-El Pardo, 28049 Madrid, Spain; ∥ The Spanish CRG Beamline, 55553 European Synchrotron Radiation Facility, 38000 Grenoble, France

**Keywords:** lithium-ion battery, ionic liquid electrolyte, transition metal dissolution, high-voltage cathode, spectroscopy

## Abstract

LiNi_0.5_Mn_1.5_O_4_ (LNMO) is a promising material for the cathode of lithium-ion batteries (LiBs); however, its high operating voltage causes stability issues when used with carbonate battery electrolytes. Ionic liquids are a viable alternative to conventional carbonate solvents due to their thermal stability and electrochemical window. This work reports the performance of LNMO/Li half cells with an ionic liquid electrolyte (ILE) composed of 0.79 molal LiFSI in trimethyl isobutyl phosphonium bis-fluorosulfonyl imide (P_111i4_FSI). The long-term stability of the cells cycled at 25 °C in ILE is superior compared to all the other cycling conditions, as shown by the Coulombic efficiency (>99.5%) and capacity retention after 210 cycles (>87.9%). Spectroscopy measurements showed that the LNMO in the LP40 cycled cells had severe structural damage, with visible holes in the surface region of the particle, extending 15–20 nm away from the surface. On the other hand, the structure of the LNMO used in the cells with ILE was similar to that of the pristine spinel after 210 cycles, the only difference being a rock-salt layer on the surface. The surface chemistry of the LNMO particles was analyzed by electron energy-loss spectroscopy and revealed that the surface region of the LNMO cycled in LP40 adopted a (Mn_*x*_Ni_*y*_)_3_O_4_-type structure in the previously described holes, while the surface chemistry was nearly unaffected by cycling in ILE. XPS highlighted the influence of the electrolyte on the nature of the cathode electrolyte interface (CEI), which showed the presence of a predominantly organic CEI after cycling in LP40. The CEI formed after cycling in ILE was thinner and dominated by species like Li_2_CO_3_ and salt decomposition products. Overall, the cycling stability of LNMO with LiFSI in P_111i4_FSI was improved, and the structural integrity was maintained with this electrolyte, as opposed to the conventional LP40.

## Graphical abstract

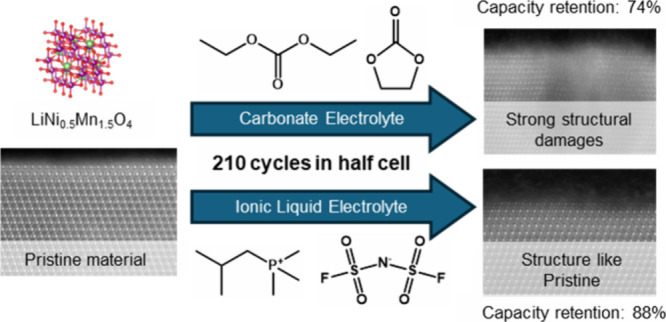

## 1 Introduction

The accelerating climate crisis pushes the development of clean alternatives to fossil fuels for energy generation and storage. Hence, the electrochemical battery industry has experienced tremendous growth in the past decade, with stationary solutions being adopted to store electricity from renewable energy production in parallel to the expansion of the electric transportation sector. Due to its high energy density, the Li-ion battery is the technology of choice, completely dominating the market for mobility. While materials like Li­(Ni_*x*_Mn_*y*_Co_*z*_)­O_2_ (NMC) and LiFePO_4_ (LFP) have been successfully used to develop batteries with high energy density and excellent lifetime, respectively, these technologies are held back by intrinsic limitations, namely the usage of the hard-to-source and unethically acquired cobalt for NMC and the lower energy density of the battery for LFP.^1,2^ LiNi_0.5_Mn_1.5_O_4_ (LNMO) is a strong alternative as cathode material, as it is Co-free and has promising electrochemical properties owing to a high operating voltage of ≈4.7 to 4.8 V vs Li/Li^+^.^3,4^ LNMO exists in two crystalline forms: the ordered spinel with a *P*4_3_32 space group and the disordered spinel with an *Fd*3̅*m* space group.^5^ As disordered LNMO shows the best properties for Li-battery applications, all of the results reported and discussed in this paper will only concern the disordered spinel.^4,6–8^ Despite its promising applications as a cathode material, LNMO faces stability issues that prevent its usage for industry-scaled battery manufacturing.

LiPF_6_ is the salt almost exclusively used in current commercial battery electrolytes. One drawback of this salt is that some HF can form due to hydrolysis following the reaction:^3,9,10^

$${\mathrm{LiPF}}_{6}+4H_{2}O\to \mathrm{LiF}+{\mathrm{PO}}_{4}H_{3}+5\mathrm{HF}$$ 1

The hydrolysis reaction 1 is due to the trace amounts of water that are always present in the electrolyte (typically 20–30 ppm). The formed HF will then react with LNMO with the hypothetical reaction, as suggested by Pieczonka et al.:^9^

$$4\mathrm{HF}+2{\mathrm{LiNi}}_{0.5}{\mathrm{Mn}}_{1.5}O_{4}\to 3{\mathrm{Ni}}_{0.25}{\mathrm{Mn}}_{0.75}O_{2}+0.25{\mathrm{NiF}}_{2}+0.75{\mathrm{MnF}}_{2}+2\mathrm{LiF}+2H_{2}O$$ 2

This process is then assumed to be the underlying mechanism behind the well-established degradation mechanism of LNMO, namely the transition metal dissolution (TMD), which is believed to be one of the primary sources of instability in LNMO-containing batteries, as the active material undergoes structural changes. Moreover, the dissolved metal can damage crucial components and accelerate the side reactions after deposition on the anode, leading to excessive growth of the solid electrolyte interphase (SEI).^11,12^ The TMD mechanism was identified by finding HF and traces of dissolved metals at the anode and in the SEI by analyzing the electrolyte and electrode surfaces with advanced techniques such as Scanning Electron Microscopy (SEM), X-ray absorption spectroscopy (XAS), X-ray photoelectron spectroscopy (XPS), nuclear magnetic resonance (NMR) and electron paramagnetic resonance (EPR).^10,13–15^ Recent studies have also shown that surface impurity groups on the cathode material, such as −OH, are suspected of participating in the TMD by catalyzing the formation of HF from the decomposition products of LiPF_6_.^7^

While salts with negligible hydrolysis compared to LiPF_6_, such as lithium bis­(fluorosulfonyl)­imide (LiFSI) and lithium bis­(trifluoromethanesulfonyl)­imide (LiTFSI), have been successfully used to reduce the TMD occurring in LiBs, especially compared to LiPF_6_, these salts fail to passivate the Al current collector above 4 V, resulting in a loss of performance over prolonged cycling.^16–20^ High-angle annular dark field (HAADF) STEM images combined with electron energy-loss spectroscopy (EELS) of pristine disordered LNMO showed a Li-depleted (Mn_*x*_Ni_*y*_)_3_O_4_-like structure at the outermost surface.^21^ Still, atomic scale resolution studies of cycled surfaces are lacking, and further study is necessary to understand the nature of the degradation. Atomic and molecular layer depositions have also been used to prevent TMD in NMC-related research and could be a potential way to improve LNMO long-term cycling.^22^

Another limitation related to LNMO stems from the use of ethylene carbonate (EC) in electrolytes. The cathode electrolyte interphase (CEI) formed by the decomposition of EC during the first charge–discharge cycles cannot withstand a potential higher than 4.4 V vs Li/Li^+^, which causes the continuous consumption of the electrolyte throughout cycling and reduces the Coulombic efficiency (CE) for each cycle.^23–26^ EC and other usual solvent components, such as dimethyl carbonate (DMC) and diethyl carbonate (DEC), also oxidize at high voltage and elevated temperatures, leading to performance loss, gas-product buildup, and other unwanted behaviors.^25,27^ It is worth noting that at high potential, the degradation of EC can lead to species that will favor the TMD, as identified by Misiewicz et al.^25^ In conventional electrolytes with LiPF_6_ and carbonates, the CEI is typically a mixture of salt reduction products (LiF,Li_*x*_PO_*y*_F_*z*_), organic compounds from carbonate decomposition,^3,17,23^ and Li_2_CO_3_.^23^ While some studies on NMC have suggested that the participation of lattice oxygens is likely responsible for the carbonated solvent oxidation,^28,29^ these mechanisms have yet to be properly identified on LNMO. Oxygen release was minor for LNMO electrodes compared to NMC electrodes in an online electrochemical mass spectroscopy study (OEMS). In fact, the release of CO/CO_2_ was similar for LNMO and electrodes made from carbon black only.^30^ In a more recent study^31^ comparing ordered and disordered LNMO, indications of loss of lattice oxygen were found to be related to anionic charge compensation. This correlation was observed more extensively for ordered materials. Finally, another disadvantage of common electrolytes is related to the flammability of the carbonated components, which has already led to significant safety issues and unwanted incidents when the battery degrades and the electrolyte catches fire.^32,33^ While some of these limits have been partially addressed by the usage of additives,^34^ more research is necessary.

Ionic liquids have recently been used as a promising alternative to the conventional carbonate-based electrolytes in LiBs. These materials are composed of cation–anion pairs with low coordination interactions and are generally in a liquid state at a temperature lower than 100 °C. Reported advantages of ionic liquids include low flammability, electrochemical stability, and nontoxicity.^35–37^ A few room-temperature ionic liquids (RTILs) solutions have been successfully used to make ionic liquid electrolytes for LiBs. A notable example is the electrolyte composed of 1.2 M LiFSI dissolved in N-propyl-*N*-methylpyrrolidinium bis­(fluorosulfonyl)­imide (PYR_13_FSI), which has allowed the fabrication of LNMO/Graphite LiBs with higher oxidative stability and better performance at elevated temperatures (45 °C) than their counterparts using a conventional electrolyte (1 M LiPF_6_ in EC:DEC 1:1, LP40). The capacity was at 81% retention after 139 cycles for the PYR_13_FSI cell compared to instant decay for the one using a conventional electrolyte.^38^ The enhanced performance of the PYR_13_FSI cells was attributed to the presence of a stable and inorganic CEI, rich in composition products from the LiFSI salt, with fewer organic products compared to the LP40 electrolyte. Similarly, Zhou and co-workers^39^ compared the degradation process between an LNMO battery cycled with LP40 electrolyte and one cycled with LiTFSI in PYR_13_TFSI and showed that the cell using the ionic liquid exhibited a significantly lower rate of dissolution for the transition metals Mn and Ni. This increased material stability resulted in better capacity retention and was attributed to a homogeneous CEI containing stable LiF species.

Trimethyl isobutyl phosphonium bis-fluorosulfonyl imide (P_111i4_FSI, Figure 1) is an RTIL with promising properties such as enhanced thermal and electrochemical stability, as well as high solubility for LiFSI salts.^40,41^ A concentration of 0.79 m was chosen, corresponding to 1.2 M, as concentrations in the range 0.5–1.0 m have been shown to be optimal for conductivity.^40^ Moreover, a solution of 0.79 m LiFSI in P_111i4_FSI has an adequate ionic conductivity of 3.8 mS cm^–1^ at 25 °C, which is sufficient for the studied charging rates despite being lower than that of LiFSI in traditional carbonate solution. Because the FSI-based ionic liquids are generally less viscous and more conductive than the TFSI-based ones, FSI is the best counterion for reaching high performance. This RTIL has been tested with batteries containing Silicon anodes and showed extended cyclability and satisfactory rate performance at cycling temperatures up to 60 °C,^42^ which was explained by the excellent conductivity, high Li^+^ transport number^,^ and SEI forming properties of P_111i4_FSI compared to other RTIL-based electrolytes such as PYR_13_FSI and PYR_13_TFSI_._ Furthermore, a 5.3 V vs Li/Li^+^ stability limit has been demonstrated for 1.2 M LiFSI dissolved in P_111i4_FSI.^42^ Despite these promising properties and cycling results, P_111i4_FSI with LiFSI salt has not yet been reported as a component for electrolytes in LNMO-containing batteries, to the best of our knowledge.

**Figure 1:**
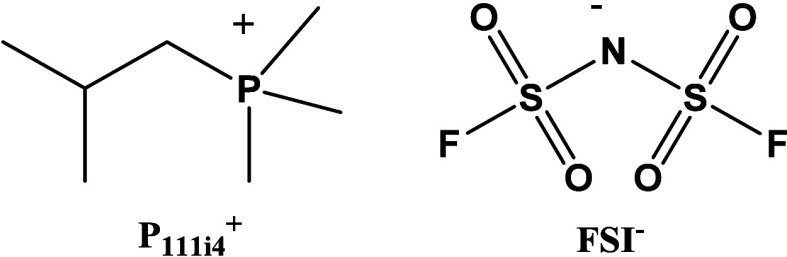
Chemical structure of P_111i_4^+^ and FSI^–^.

In this work, the electrochemical properties and degradation mechanisms of LNMO/Li half-cells with an electrolyte composed of 0.79 molal LiFSI in P_111i4_FSI are reported and compared to those of the same half-cell with a conventional LP40 electrolyte. The morphology and chemical properties of the cell before and after cycling were investigated with a combination of scanning electron microscopy (SEM), focused ion beam (FIB), transmission electron microscopy (TEM), atom probe tomography (APT), X-ray photoelectron spectroscopy (XPS), and hard X-ray photoelectron spectroscopy (HAXPES). This study aims to enhance the understanding of LNMO electrode degradation after cycling in these electrolytes by examining atomic-scale structural changes and analyzing the composition of the resulting CEI layer, regardless of the anode’s nature.

## 2 Experimental Methods

### 2.1 Electrode Preparation

Electrodes were prepared by mixing 90 wt % nonstochiometric LiNi_0.43_Mn_1.57_O_4_ (Haldor Topso̷e, Denmark), 5 wt % polyvinylidene fluoride (PVDF) (Arkema Kynar HSV 900, France) binder, and 5 wt % carbon black (Imerys, C-NERGY C-65, France) in 1-Methyl-2-pyrrolidinone (NMP) (Thermo Fisher Scientific, USA). LNMO and carbon black were mixed for 2 min at 25 Hz with a Retsch Mixer MM400 shaker mill in ZrO_2_-coated shaker jars together with three ZrO_2_ balls, then liquids were added, and the slurry was mixed for 30 min at 25 Hz. Electrode foils were prepared by tape casting the slurry with a loading between 0.18 and 0.23 mAh cm^–2^ onto a carbon-coated Al foil (MTI, China) and drying overnight at 60 °C. While this loading is lower than typical industrial values (∼2.5 to 4 mAh cm^–2^), it is considered sufficient to study the degradation mechanism of the active material. The electrodes were then calendared, targeting a thickness between 10 and 12 μm. Lastly, electrodes were cut to a diameter of 14 mm and dried at 110 °C under an active vacuum before transfer into the glovebox (MBraun Labmaster, H_2_0 < 0.1 ppm, O_2_ < 0.1 ppm).

### 2.2 Galvanostatic Cycling in Half Cells at 25 and 45 °C

Half cells were chosen as the primary mode of investigation because they provide an almost infinite amount of lithium for cycling, allowing the degradation of the battery to be focused mainly on the cathode. Half cells were prepared in small pouches with the LNMO electrodes as the positive electrode and Li metal (16 mm Ø) as the negative electrode. The half cells using carbonated electrolyte were cycled with 40 μL 1 M LiPF6 in EC:DEC (50/50 v/v) (Battery grade, Sigma-Aldrich) and one Celgard 2325 separator (25 μm), while the cells with the ILE used 80 μL of 0.79 m LiFSI in P111i4FSI with a glass fiber separator (Whatman, 260 μm) sandwiched between two alumina-coated PP/PEs separators (Freudenberg, 25 μm). Two formation cycles were performed at C/10, and subsequent cycling consisted of 50 C/2 cycles followed by two C/10 cycles, repeated four times, which resulted in a total of 210 cycles. Both electrolytes were tested at 25 and 45 °C.

### 2.3 Transmission Electron Microscopy and Scanning Transmission Electron Microscopy

Specimens for TEM investigations were prepared by using a dual-beam SEM-FIB (FEI Helios G4 UX, USA). A carbon layer was initially deposited to protect the surface of the LNMO particles, and final thinning was performed with the ion beam accelerated to first 5, then 2 kV.

TEM investigations were performed using an aberration-corrected microscope with a cold-field emission gun operated at 200 kV (Jeol JEM ARM 200F, Japan). The instrument was equipped with a Quantum GIF Dual EELS for electron energy-loss spectroscopy (EELS). EELS was performed with an incident beam semiconvergence angle of 20 mrad and a collection angle into the GIF of 67 mrad. The step size was 1.5 or 2 Å, the pixel time was 0.01 s, and the EELS energy resolution was 0.25 eV/ch. All spectroscopic data were acquired over an area of approximately 40 nm × 40 nm for a short time and integrated perpendicular to the surface of interest to reduce noise and avoid beam damage.

Compositional analysis based on EELS was performed using Gatan Digital Micrograph (version 3.4.3). The background was fitted with a power law, the energy-loss near-edge structure (ELNES) was excluded, and the Hartree-Slater Cross-section was used. Plural scattering was accounted for by utilizing the high- and low-loss spectra. The Mn fine structure was analyzed using HyperSpy v. 1.7,^43^ where background subtraction was performed using the power law. The beam semiconvergence angle was 27 mrad, and the collection angles of the HAADF-STEM images were taken from 51 to 203 mrad. Lastly, the SmartAlign plug-in^44^ was utilized to obtain high-quality structural images. Twenty images were obtained with a pixel time of 2 μs, and a 90° scan rotation was applied between each image.

### 2.4 Atom Probe Tomography

Specimens for APT were prepared following a site-specific lift-out protocol described by Thompson et al.^45^ An FEI dual beam Helios G4 UX was used for the APT sample preparation. The APT specimens were analyzed using a Cameca LEAP 5000 XS instrument operating at 15 pJ laser energy, 250 kHz, and 50 K. The commercial package AP Suite 6.3.1 was used for data reconstruction and analysis.

### 2.5 X-ray Photoelectron Spectroscopy

The cathodes were rinsed with dimethyl carbonate (DMC; ≥99%, Sigma-Aldrich) and transferred for measurement in an Ar-filled transfer chamber. A Kratos Axis Ultra with an electrostatic/magnetic hybrid lens was used for the XPS measurement, with an Al–Kα radiation at 1486.6 eV. The measurements were recorded with an emission current of 10 mA and an operating accelerating voltage of 12 kV. The samples were mounted on electron-conducting carbon tape on a copper stub. The C 1s, O 1s, F 1s, P 2p, S 2p, N 1s, Mn 2p, and Ni 2p regional spectra were collected with a 20 eV pass energy. The spectra of the different materials were aligned using the C–C peak at 284.8 eV. The different peaks used to fit the data were a combination of Lorentzian and Gaussian functions (50:50) calculated by fixing constraints on the area ratios, positions, and full width at half-maximum of the different components, following reported data and the chemical structure of the component.

### 2.6 Hard X-ray Photoelectron Spectroscopy

The HAXPES samples were collected and transferred in the same way as the XPS samples. The measurement was performed at the European Synchrotron Radiation Facility at the Spanish CRG BM25-SpLine beamline with a Cylindrical Mirror Analyzer (HV-CSA300). A D25 magnet delivered X-ray photons at 7 keV that were monochromatized by a Double Crystal Monochromator. The beam was focused on the center of the different samples with a footprint of 300 × 700 μm^2^. The samples were kept in an Ar atmosphere before being transferred to the analysis chamber. A reference Au standard was used to calibrate the binding energy of the different samples.

## 3 Results and Discussion

### 3.1 Battery Performances

The average specific discharge capacities and Coulombic efficiencies (CE) obtained during the cycling of three LNMO half-cells are presented in Figure 2a,b. The shaded region indicates the standard deviation obtained from three different half cells, which is quite large for the discharge capacity, exhibiting the well-known issue of reproducibility observed in Li-ion research. Still, the trends were consistent between repeats, and the average value should be representative of the general electrochemical behavior of the different cells.

**Figure 2:**
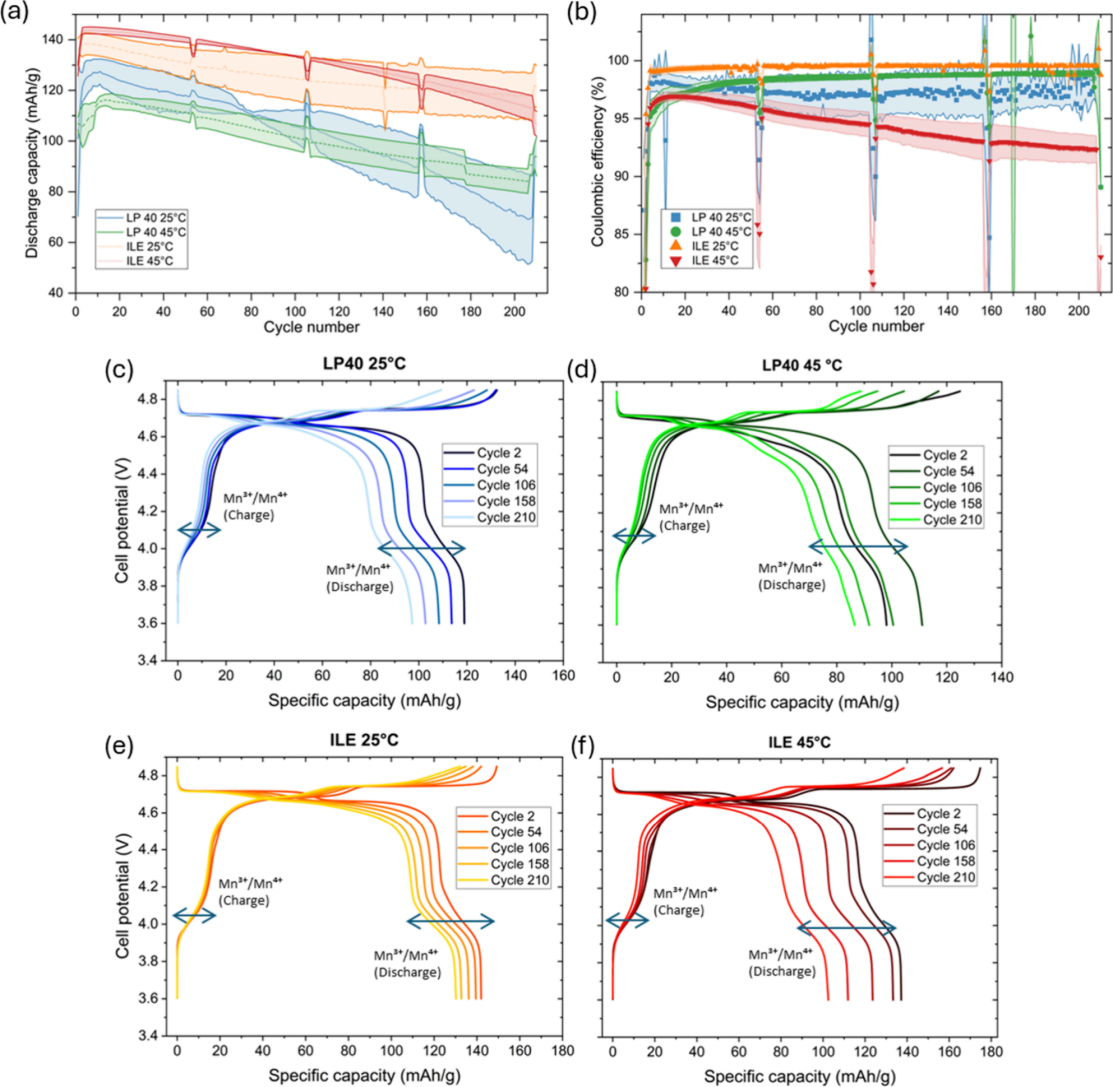
(a) Specific discharge capacity and (b) Coulombic efficiency for LNMO cycled with LP40 and ILE at 25 and 45 °C for 210 cycles. Voltage profile as a function of specific capacity for the second C/10 cycles of the best-performing cells using (c, d) LP40 and (e, f) ILE at 25 and 45 °C, respectively.

The cells cycled with ILE deliver a high initial discharge capacity at both cycling temperatures, with starting values close to the theoretical maximum discharge capacity of 147 mAh g^–1^, while the cells using LP40 start at values <130 and <120 mAh g^–1^ at 25 and 45 °C, respectively (Figure 2a). The long-term stability of the cell cycled at 25 °C in ILE is superior compared to all the other cycling conditions, as shown by the high CE (>99.5%, Figure 2b) and capacity retention after 210 cycles (87.9%). On the other hand, LP40 at 25 °C, LP40 at 45 °C, and ILE at 45 °C had capacity retentions of 73.7, 77.8, and 74.3%, respectively. The voltage profiles as a function of the specific capacity for every second C/10 cycle (Figure 2c–f) highlight the influence of the electrolyte and cycling temperature on cell stability. The electrode cycled in ILE at 25 °C shows remarkable stability during charging, as it is the only cell where the plateau associated with the Mn^3+^/Mn^4+^ transition is mostly constant over the whole cycling (Figure 2e). On the other hand, this plateau changes in shape for the LP40 cells (Figure 2c,d) and the ILE 45 °C battery (Figure 2f), indicating that the electrochemical environment surrounding Mn in these materials could be changing during cycling. The Mn^3+^/Mn^4+^ discharge plateau of the LP40 45 °C cell (Figure 2d) changes drastically during cycling, suggesting strong damage to the LNMO structure.

For the LP40 cells at 45 °C, the capacity of the initial cycles is quite low when compared to the other electrolytes. This capacity increases and reaches its maximum value at cycle 10. This behavior suggests that elevated temperatures hinder initial performance and may delay the formation of a stable CEI by a few cycles for the LP40 electrode, as indicated by the low Coulombic efficiencies. It should be noted that all cycles, including the formation at C/10, were conducted at 45 °C for the high-temperature cycling. This implies a nonoptimized formation protocol, and cycling at elevated temperatures is mainly included for post-mortem analysis, as described later.

For the 45 °C ILE cell, the CE is low and varies between 97% and 93% during most of the cycling, indicating severe side reactions at elevated temperatures. The voltage profiles show an extended plateau at around 4.75 V, which appears to correlate with the lower CE. The lower discharge capacity observed at the slow cycles is attributed to the presence of side reactions. The combination of slow charge, and hence long time spent at highly oxidizing potentials, and elevated temperatures appears to be detrimental to the electrode performance.

While the high operating voltage raises concerns about the potential corrosion of the Al current collector, cyclic voltammetry of aluminum paper with a thin layer of carbon (Supporting Information, Figure S1) has shown current densities lower than 3.5 and 8 μA cm^–2^ for LP40 and ILE, respectively. Hence, it can be assumed that Al corrosion is insignificant in the studied systems.

### 3.2 SEM Analysis

The SEM images of the surfaces and cross-section of representative particles before and after cycling (Figures S2 and S3, Supporting Information) show that for the LNMO electrode cycled in the ILE at room temperature, particles appear to conserve most of their integrity after cycling and can hardly be distinguished from the pristine electrode. Conversely, cracks can be observed on the particle cycled in ILE at 45 °C at the grain boundaries (Figure S3c,d), as indicated by the yellow arrows. These cracks may expose fresh surfaces for side reactions, which could be the reason for the lower CE. No visible damage is observed on the surface of the particles cycled in ILE at 45 °C. For the LP40 samples, some small damage is visible on the edges of the LNMO crystals, which is to be expected from the TMD related to HF formation when LiPF_6_ reacts with the trace amount of water.

### 3.3 STEM Analysis

The HAADF-STEM images (Figure 3) acquired from the pristine and cycled materials exhibit the impact of the electrolyte’s nature and the cycling temperature on the structure of LNMO near the surface of the particle. The high-quality pristine material is shown in Figure 3a,b, where the majority of the structure is dominated by the LNMO-spinel, except the upper ∼1.5 nm, which has an (Mn_*x*_Ni_*y*_)_3_O_4_-like structure.^46^ Figure 3k shows the LNMO spinel structure, with the Oxygen atoms omitted. When LNMO transforms into (Mn_*x*_Ni_*y*_)_3_O_4_, the transition metals (TM) insert into the Li positions like (Mn/Ni)_Li_ antisite defects.

**Figure 3:**
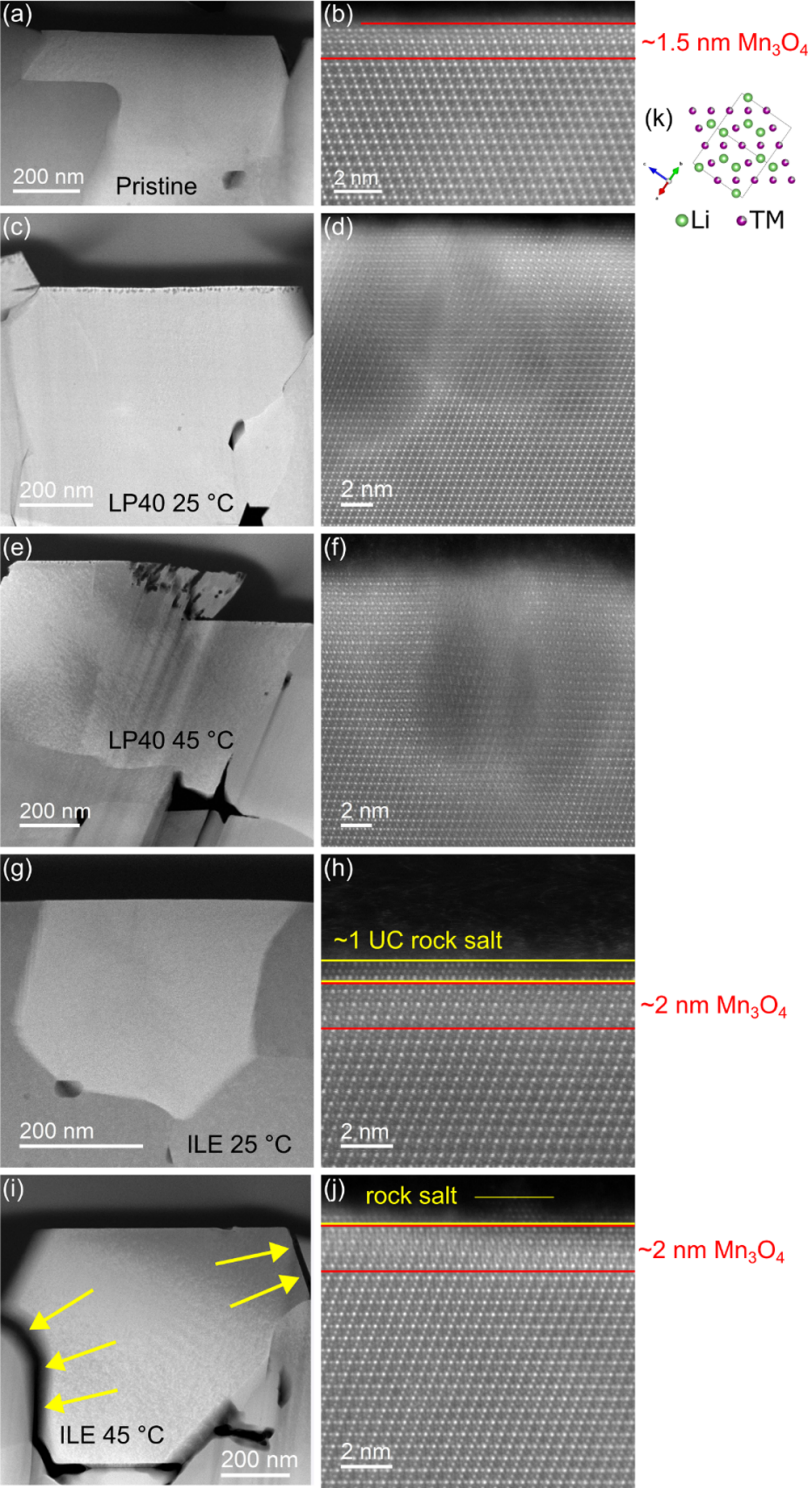
HAADF-STEM images of an LNMO particle before cycling (a, b), after cycling in LP40 at 25 °C (c, d) and 45 °C (e, f), and after cycling in ILE at 25 °C (g, h) and 45 °C (i, j). Arrows in (i) indicate cracking along grain boundaries. (k) LNMO spinel structure with the oxygen atoms 21 omitted. Li is invisible in HAADF-STEM images, but for the (Mn_*x*_Ni_*y*_)_3_O_4_ layers, TMs occupy the Li sites in the structure*.* ^46^

LNMO particles cycled with LP40 look structurally similar at the nanoscale at both cycling temperatures, and holes are visible along the surface (Figure 3c,e). The holes reach about 15 nm into the structure, while a high degree of crystallinity is maintained in the near vicinity of the holes, as demonstrated by the Fast Fourier Transform (FFT)’s taken at two different areas in Figure 3d,f, which is shown in the Supporting Information (Figure S4). Some important structural damages, looking like pits, can be observed in Figure 3e,f, which are consistent with the TMD mechanism.^47,48^

When cycled in ILE, LNMO mostly retains its atomic structure in the near-surface region (Figure 3h,j). However, the previously observed (Mn_*x*_Ni_*y*_)_3_O_4_ phase extends slightly deeper into the material than the pristine electrode, with a 2 nm thickness. A layer of approximately one unit cell of rock-salt structure can be observed on the outermost border of the particle. The formation of (Mn_*x*_Ni_*y*_)_3_O_4_ -surface layers and rock-salt layers is well-known for both layered oxides and spinel cathode materials, and is attributed to the migration of TM cations into tetrahedral sites (Mn_*x*_Ni_*y*_)_3_O_4_ and octahedral sites (rock salt).^49,50^ The surface layer of (Mn_*x*_Ni_*y*_)_3_O_4_ is also observed in pristine LNMO of the same type as the material used here.^21^ It has furthermore been established that (Mn_*x*_Ni_*y*_)_3_O_4_ dissolves in carbonate electrolytes, as shown for 1.2 M LiPF6 in EC/EMC (3:7) electrolyte,^51^ and at the same time, it will reform during charging.^50^

These results suggest that cycling in the ILE has a relatively low impact on the crystalline structure of the LNMO and that the rock-salt layer does not spread deeper into the particle, regardless of the cycling temperature. While excessive growths of rock-salt layers are frequently reported to be detrimental for layered oxides, such layers have also been proposed to prevent the TMD in spinel and layered oxide cathode materials. However, concerning the results presented here, it cannot be verified whether the lack of structural damage is mainly related to the absence of TMD in the ILE or if it is prevented by the rock-salt layer.^52,53^

Conversely, the cracking damages observed on SEM at elevated temperatures on the cells cycled in ILE can also be observed on the HAADF-STEM images when comparing Figure 3g,i (as shown by arrows), suggesting some potential degradations at the grain boundary. These cracked particles would also have a higher surface area than the intact LNMO, which could explain the lower CE when cycling at elevated temperatures, as more CEI would need to be formed at the interface with the electrolyte.

### 3.4 EELS and APT Measurements

EELS was performed to study the variation in O, Mn, and Ni content in the subsurface layers of LNMO up to 35 nm depth­(Figure 4). Since Li is difficult to detect and measure quantitatively using EELS, O, Mn, and Ni were measured using the high-loss spectrum, and the quantification procedure assumes that the sum of O, Mn, and Ni equals 100% of the concentration. It should also be noted that the use of Hartree-Slater cross sections for EELS quantification is subject to relative errors in the range of 10–20%,^54^ however, the analysis still provides valuable insight into the chemical variation in the subsurface layers of LNMO cycled at different conditions.

**Figure 4:**
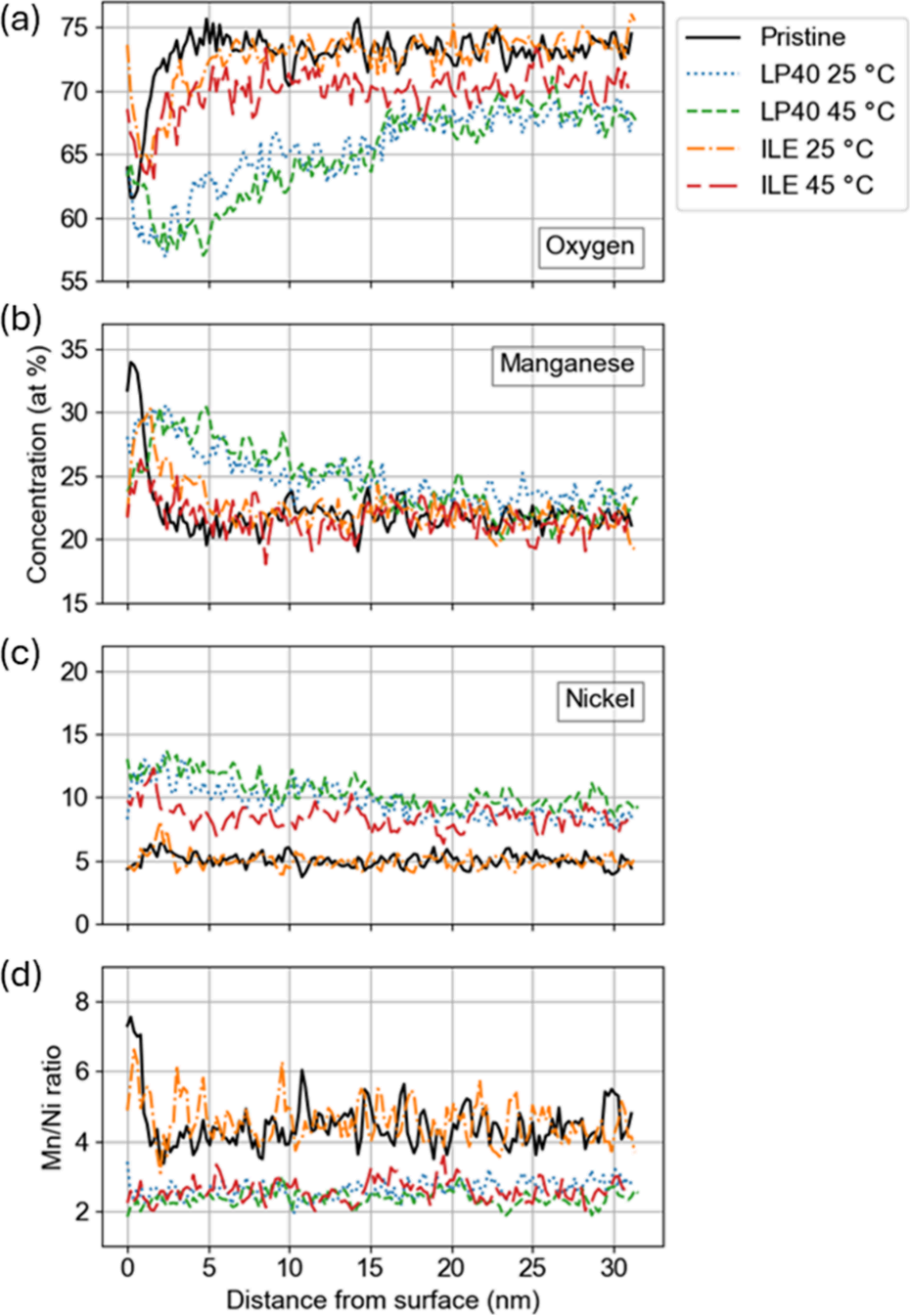
Concentrations of (a) O, (b) Mn, and (c) Ni measured by EELS and plotted as a function of distance from the surface. (d) Mn/Ni ratio is plotted for all conditions.

For the pristine LNMO, the concentration of the elements in the outer ∼3 nm from the surface differs from the bulk concentrations, with an apparent increase in Mn and a decrease in O, while Ni appears constant. These results are consistent with the (Mn_*x*_Ni_*y*_)_3_O_4_-phase on the surface of the grain, as previously observed with HAADF-STEM (Figure 3a,b).

The structural damage for LNMO cycled with LP40 also affects the chemical composition of the grain, as a relative loss of lattice O and increases of Mn and Ni can be seen from the surface to a depth of around 20 nm. At a depth of approximately 3 nm, the O, Ni, and Mn contents in LP40 are about 57, 30, and 13%, respectively. These proportions align with what you’d expect for an (Mn_*x*_Ni_*y*_)_3_O_4_ spinel phase and imply that the disrupted LNMO, specifically, the freshly exposed surfaces inside the pits, adopts a chemistry similar to that of the surface of the pristine LNMO. This is consistent with the previous reports on dissolution of the (Mn_*x*_Ni_*y*_)_3_O_4_, and reformation of the same phase upon charging.^50,51^ Then, the O and Mn concentrations gradually change and stabilize at ∼67 and 23% (Figure 4a,b), respectively, from 22 nm deepness, while Ni only slightly decreases to ∼9% (Figure 4c), which is different from the ∼5% Ni observed in the pristine LNMO. The cycling temperature does not seem to impact the structure strongly when cycling in LP40, with the only noticeable difference being a slightly higher O concentration in the 3–22 nm depth range for the sample cycled at 25 °C (Figure 4a). This chemical variation is present in the same region as the observed holes (pits) in Figure 3d,f, with a similar depth of ∼22 nm. Overall, it is very likely that these damages are caused by the TMD.

The influence of the cycling temperature is much more apparent for the ILE samples, as at 25 °C the O, Mn, and Ni concentration profiles are very similar to that of the pristine LNMO, while at 45 °C the O and Ni are respectively lower and higher than the pristine material through the 2–30 nm layer of the depth profile. These changes highlight the positive effect of the ILE electrolyte on the stability of the LNMO structure while also exhibiting the negative influence of an increased cycling temperature. Still, the structural damages are less pronounced than in the LP40 case, even at 45 °C, and no (Mn_*x*_Ni_*y*_)_3_O_4_-phase is observed within the LNMO particle. There are substantial changes at the surface (0 nm depth) of the ILE materials, with an O concentration of up to ∼74%, which is most likely due to the formation of an O-rich CEI layer when cycling in ILE, as confirmed by XPS later in this study (Figure 6).

The Mn/Ni ratio (Figure 4d) is constant for all materials from ∼1 nm depth. For the ILE 25 °C electrode, the ratio is practically similar to the pristine electrode, confirming the structural integrity of the electrode, even after more than 200 cycles. The ratio is lower than the pristine for the LNMO cycled in LP40 at both temperatures and for the ILE 45 °C sample. This difference indicates that LNMO suffers a greater loss of Mn compared to Ni when cycled with these conditions, which could be explained by the Mn-disproportionation reaction typical of LMO and LNMO materials.^55,56^ This result is also consistent with the voltage profiles (Figure 2c–f), where the Mn^3+^/Mn^4+^ plateau almost disappears for both LP40 electrodes and the ILE 45 °C electrode. At the same time, it remains constant over the cycles for the ILE 25 °C cells.

APT was performed on LNMO cycled 40 times in LP40 at 25 °C to investigate the evolution of Li concentration near the surface of the particle (Figure S5, Supporting Information). The concentration profiles of Mn, Ni, and O follow the same behavior as observed by the EELS analysis. In addition, APT reveals that the Li concentration is lower up to 7 nm deep from the surface, exhibiting the loss of this element after cycling, which is different from the pristine material, which only shows low Li concentration up to 1.5 nm depth due to the surface (Mn_*x*_Ni_*y*_)_3_O_4_-layer. In this context, the APT qualitatively supports the profiles obtained for Mn, Ni, and O. It should be noted, however, that neglecting the Li-concentration in the analysis (O + Mn + Ni = 100%) is a better approximation near the surface than in bulk and might be the reason for the slight shifts in the bulk concentrations.

The fine structure analysis (Figure 5) demonstrates that the pristine and cycled materials follow the same trend of a lower Mn oxidation state at the surface and a higher Mn oxidation state in the bulk. Similarly to the structural and chemical variation, the oxidation state only changes in the outer few nanometers for the pristine material. For LNMO cycled with LP40, the oxidation state is different from that of the pristine LNMO up to ∼22 nm into the material. It shows a distinct step-behavior at ∼13 nm, which is also present in the O-concentration profile (Figure 4a) and is typical of the (Mn_*x*_Ni_*y*_)_3_O_4_-phase. Hence, it is suggested that the structure at the surface of the pits previously observed with HAADF-STEM is of the same nature as the surface of the uncycled LNMO.

**Figure 5:**
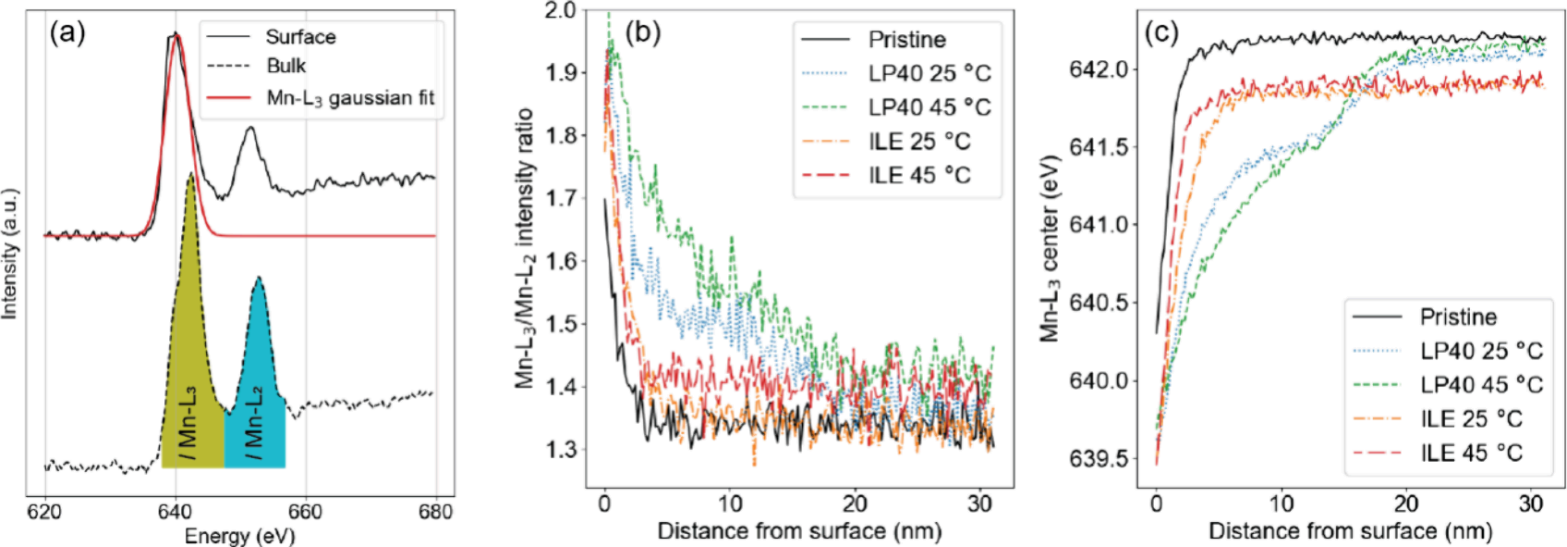
(a) Mn EELS spectra taken from the surface and the bulk of the pristine material, illustrating the Gaussian fit and numerical integration procedure. The calculated intensity ratios and fitted peak positions are shown for all the cycling conditions in (b) and (c), respectively.

The behavior of the Mn oxidation state in ILE at 25 and 45 °C is again much more similar to that of the pristine material since it only varies from bulk in the outer few nanometers (Figure 5b,c). The slightly lower position of the Mn-L_3_ center beyond 3 nm compared to the other materials is most likely due to a small shift of the zero-loss peak used for calibration, a common occurrence in EELS. After the first few nm, the Mn-L_3_/Mn-L_2_ ratio of ILE at 45 °C seems to stabilize at a slightly higher level than ILE at 25 °C and the pristine material, meaning that the Mn oxidation state is slightly lower for ILE at 45 °C. It is hypothesized that this might be due to the added degradation caused by cycling ILE 45 °C as indicated by the cracking of the grain boundaries near the surface, slightly lower O content as shown by EELS in Figure 4a, as well as the unstable CEI formation described through the analysis of the XPS data in Section 3.5.

Overall, the stability of the voltage profiles, combined with the structural and compositional investigations of the cycled LNMO surface by HAADF-STEM and EELS, demonstrate that the TMD is almost negligible upon cycling in the ILE electrolyte.

### 3.5 XPS and HAXPES Analysis

The proportion of each element on the surface of the cathode was calculated by XPS using the average of 5 survey scans and the quantification method of the software CASA XPS (Figure 6). The proportion of the surface elements changes from the pristine condition after cycling for all samples, suggesting the presence of a CEI and some changes to the original cathode chemistry.

**Figure 6:**
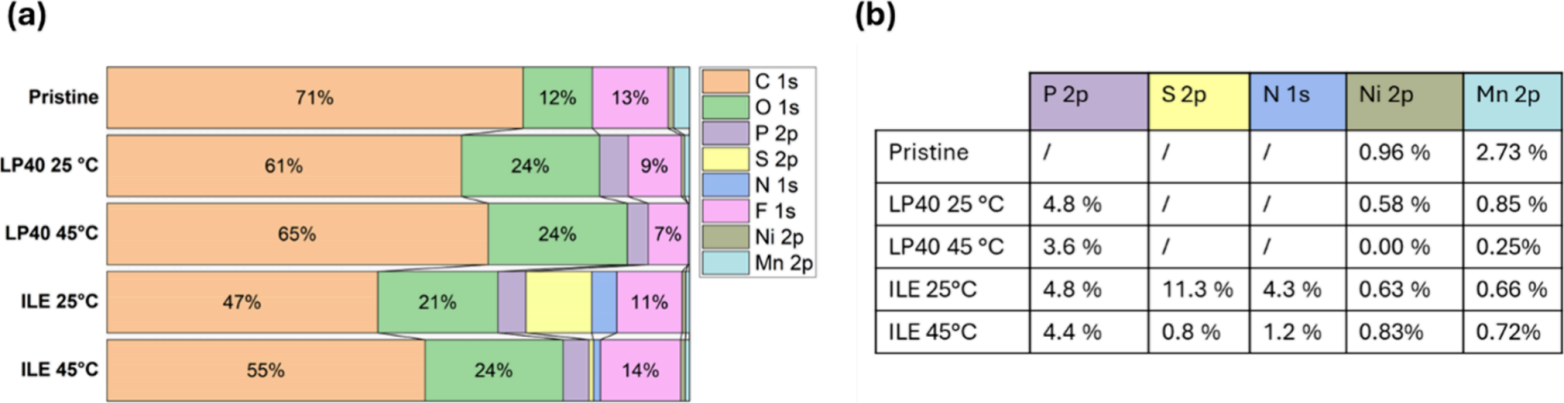
(a) XPS quantification of the pristine LNMO cathode and the cathode after cycling at different temperatures and with different electrolytes, with (b) table detailing the quantification of P 2p, S 2p, N 1s, Ni 2p, and Mn 2p.

XPS analysis of the pristine material and the cathode of the half-cells cycled in LP40 or ILE at 25 °C (Figure 7, Table 1), the cells cycled at 45 °C (Figures S6 and S7, Supporting Information), and the electrodes kept at OCV for one month (Figures S8 and S9, Supporting Information) was conducted to understand the nature of the CEI and the influence of cycling and temperature on the degradation process. All results using XPS are only applicable to the surface of the material up to 5∼10 nm depth, as the penetration of the X-rays is limited.^57^

**Table 1:** Summary of Chemical Links Identified with XPS

	C 1s	O 1s	F 1s	P 2p	S 2p	N 1s
Pristine	C–C, CH_2_, C–F_2_, C=O, C–O/C–H	Metal Oxide, C=O/CO_3_, C–O	C–F	/	/	/
LP40 25 °C	C–C, CH_2_, C–F_2_, C=O, C–O/C-H, O=C–O/–CO_3_	Metal Oxide, C=O/CO_3_/P=O, C–O, O=C–O	C–F, P–F, Li–F	/		/
LP40 45 °C	C–C, C=O, C–O/C–H, O=C–O/–CO_3_	Metal Oxide, C=O/CO_3_/P=O, C–O, O=C–O	P–F	/	/	/
ILE 25 °C	C–C, C=O, C–O/C–H, O=C–O/–CO_3_	Metal Oxide, C=O/CO_3_/P=O, C–O	S–F, Li–F	P_111i4_, *P*=O	FSI/S–N, S–F	FSI/S–N, Li–N
ILE 45 °C	C–C, CH_2_, C–F_2_, C=O, C–O/C–H, O=C–O/–CO_3_	Metal Oxide, C=O/CO_3_/P=O, C–O	C–F, S–F, Li–F	*P*=O	S–F	Li–N

**Figure 7:**
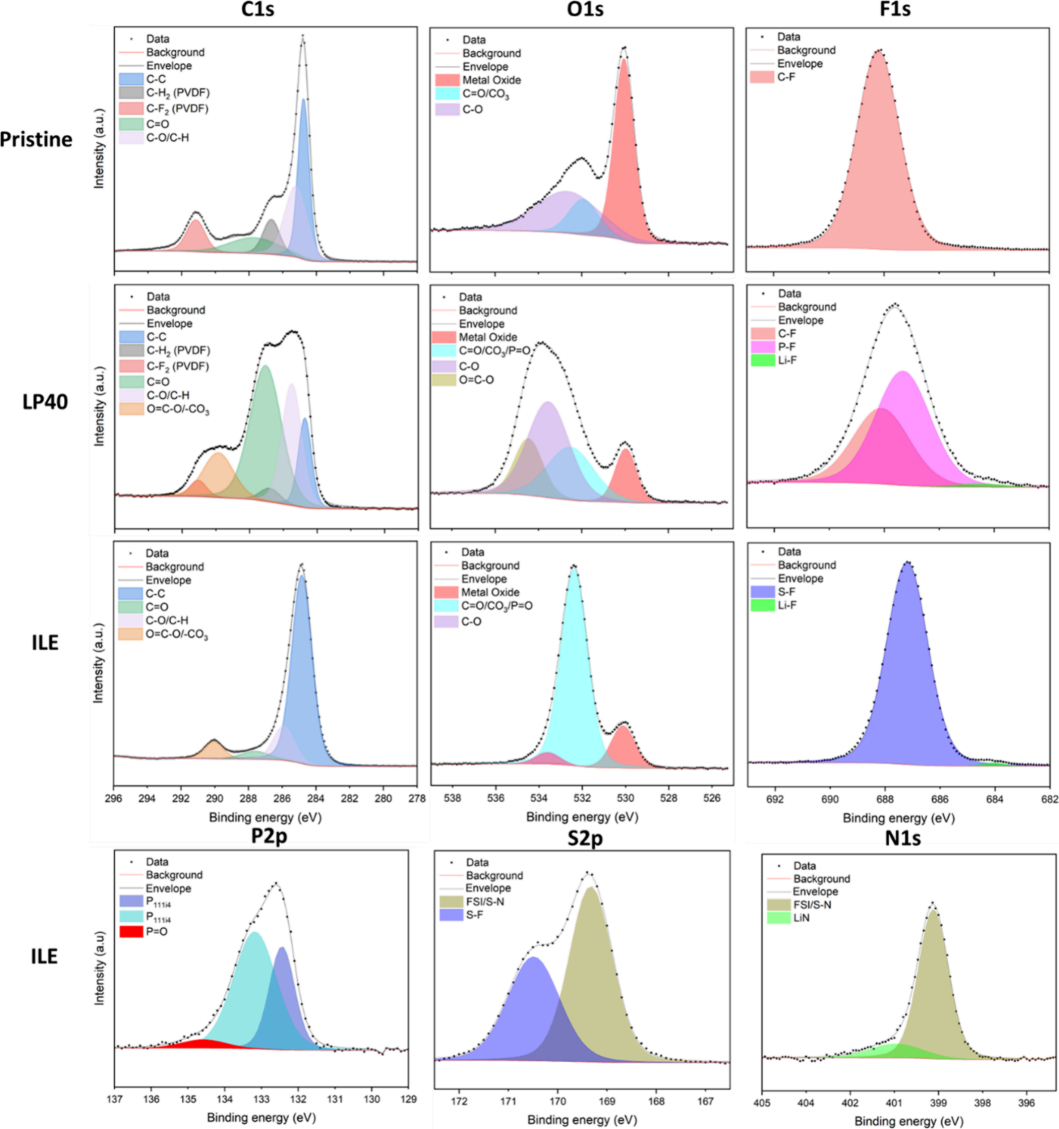
Deconvoluted XPS spectra of C 1s, O 1s, F 1s, P 2p, S 2p, and N 1s of the pristine LNMO cathode and the LNMO cathode cycled for 200 cycles in LP40 or ILE at 25 °C.

The pristine and 25 °C samples (Figure 7) showed the same strong C–C peak at 284.8 eV on the C 1s spectra (blue), primarily attributed to the carbon black with some added contribution from the PVDF binder. The polymer binder is also responsible for the C–H_2_ (gray) and C–F_2_ (red) peaks observed at 287.0 and 291.1 eV on the C 1s of the pristine sample, respectively.

C=O (green) and C–O/C–H (light pink) peaks are observed on all samples at 287.8 and 285.4 eV, respectively, on the C 1s spectra. These peaks are also present on the O 1s spectra of every material at 531.5 and 532.2 eV, respectively, with some added contribution from eventual CO_3_ and P=O groups. An O=C–O (light yellow) peak appeared on the LP40 sample at 534.2 eV on the O 1s spectrum, which is also observed as part of the O=C–O/–CO_3_ contribution at 290.1 eV (light orange) on the C 1s spectrum. The high amount of carbon–oxygen and carbon–hydrogen bondings on the LP40 cathode indicates the formation of a CEI rich in organic compounds. These contributions are still present for LP40 45 °C (Figure S6, Supporting Information), and it can be assumed that the nature of the CEI is not influenced by the cycling temperature. However, the reduction of the C–C feature means that the upper layer formed by the electrochemical reaction is getting thicker, and that the increased temperature accelerates the CEI formation reaction. The presence of a thicker upper layer is also supported by the decrease of the metal oxide peak at 530.1 eV on the O 1s (light red) between LP40 25 °C and LP40 45 °C. For the ILE electrolyte, the CEI appears to be thinner, based on the C–C and the N 1s peak after 200 cycles. Also, for the ILE electrolyte, the reduction of these peaks indicates a growth in the CEI thickness. This is in line with the observed differences in polarization seen from the voltage profiles in Figure 2, with the lowest polarization observed for ILE at 25 °C, and the highest for LP40 at 45 °C. On the ILE side, there is no O=C–O feature on the O 1s spectra, meaning that the contribution at 290.1 eV on the C 1s (light orange) is most likely due to the presence of Li_2_CO_3_ groups. These species are also confirmed by the substantial contribution at 532.2 eV on the O 1s (bright blue), which is most likely related to the CO_3_ groups and not much to P=O, as this feature is small on the P 2p side (bright red). The increased cycling temperature had a noticeable effect on the C 1s and O 1s features, with the contributions related to C–O, C–H, and O=C–O increasing on the ILE 45 °C cathode.

The differences of intensity for the C–O/C–H peaks observed between the C 1s spectra of the ILE OCV samples at 25 and 45 °C (Figure S8, Supporting Information) show that, in ILE, the increased temperature influences the chemical structure around the carbon element at the cathode’s surface, even without electrochemical cycling, which could indicate that some of the organic species are thermally unstable. On the other hand, the Mn 3p and Ni 2p spectra of the OCV LP40 sample kept at 25 °C are different from those of the sample kept at 45 °C, and they are the same for the ILE OCV samples kept at 25 and 45 °C (Figure S9, Supporting Information). These results indicate that LP40 could react with the LNMO particle, even without electrochemical cycling.

The F 1s spectra of the LP40 and ILE 25 °C samples both show a minor Li–F feature (bright green) at 683.7 eV. Similarly to the results for the C 1s spectra, the C–F peak of the PVDF (red) is observed at 687.3 eV in the F 1s spectra of both the Pristine and LP40 cathodes, while absent from the ILE sample. The P–F feature (bright pink) at 686.9 eV observed on the LP40 electrode suggests that some of the LiPF_6_ salt could still be present on the surface of the cathode or that the CEI incorporated it into its chemical structure. Alternatively, it could be related to the presence of Li_*x*_PF_*y*_O_*z*_, a typical CEI decomposition product.^16^ Similarly, the F 1s of the ILE show an S–F feature at 686.7 eV, which could indicate that LiFSI salts remained at the cathode and that there are F–S chemical bondings in the CEI. The presence of these features is also consistent with the presence of both FSI and S–F features on the S 2p spectrum of the ILE at 168.9 and 170.0 eV, respectively. Remnants of LiFSI salt are identified in the N 1s spectrum of ILE at 399.1 eV. This spectrum also shows a Li–N feature (faint purple) at 401.0 eV. When comparing the shape of the P 2p, S 2p, and N 1s of the ILE sample cycled at 25 °C (Figure 7) and 45 °C (Figure S7, Supporting Information), it is evident that the contributions attributed to FSI and P_111i4_ are not present at elevated temperature. The decomposition products of these species may play a key role in forming an electrochemically stable CEI, which is unstable at elevated temperatures and needs to be reformed each cycle.

A substantial increase in O content from 12 to 24% compared to the pristine material could indicate CEI formation by EC and DEC decomposition. The preferential loss of Mn compared to Ni observed by EELS (Figure 4d) is also detected by XPS at 25 °C, as the Mn/Ni ratio goes from 3.85 on the surface of the pristine LNMO to 2.47 on the surface of the LP40 cycled sample. The increased temperature appears to result in the formation of a thick CEI on the surface of LNMO, with the Ni completely disappearing after cycling at 45 °C in LP40 and the Mn being reduced to only 0.25%. The temperature also appears to influence the decomposition of the carbonate species in LP40, as indicated by the increase in C content, accompanied by a reduction in the C–C peak. Some P can be observed on the cathode cycled in LP40, which could be either LiPF_6_ salt residue or part of the CEI, i.e., Li_*x*_PF_*y*_O_*z*_.^16,58,59^

Concerning the ILE sample cycled at 25 °C, the significant presence of P, S, and N at 4.8, 11.3, and 4.3%, respectively, and the increase of O content to 24% suggest that both LiFSI and P_111i4_FSI are either reacting to form the CEI or are still present on the surface, even after washing with DMC. The cycling temperature strongly affects the chemistry of the surface, as the amounts of S and N are reduced to 0.8 and 1.2%, respectively, after cycling at 45 °C. The P content is relatively constant at 4.4% at 45 °C, and the C content increases from 47% at 25 °C to 55% at 45 °C. The S and N loss could mean that the species formed from FSI^–^ are unstable at elevated temperatures, while the constant P content suggests that those containing P_111i4_ are more thermally stable.

When comparing the Mn 2p and Ni 2p (Figure 8a–c) of the pristine sample (black line) to these of the LP40 cycled cathode (red line), it appears that most of the typical Mn peaks at 642.2 and 654.0 eV and Ni peaks at 854.6, 860.5, and 872.1 eV are either strongly attuned or absent after cycling. This attenuation could indicate that the LNMO particles were significantly damaged by the cycling, causing the loss of the typical metallic features. This assumption is supported by the HAADF-STEM and EELS of the LP40 samples, which showcase extensive damage to the structure of the material at and close to the surface. Moreover, the total loss of the sharp Ni 2p_2/3_ peak^60^ at 854.6 eV on the Ni 2p spectrum indicates that the crystalline and chemical structure of LNMO is likely not intact after cycling with LP40. On the other hand, the typical Mn and Ni peaks of LNMO are still present after cycling in ILE, as shown by the Mn 2p and Ni 2p spectra (blue line). There is only a slight decrease in intensity compared to the pristine electrode, confirming that the upper part of the LNMO particles is mostly intact. This result confirms the conservation of the particle integrity when cycling in ILE, as previously observed with HADF-STEM and EELS.

**Figure 8:**
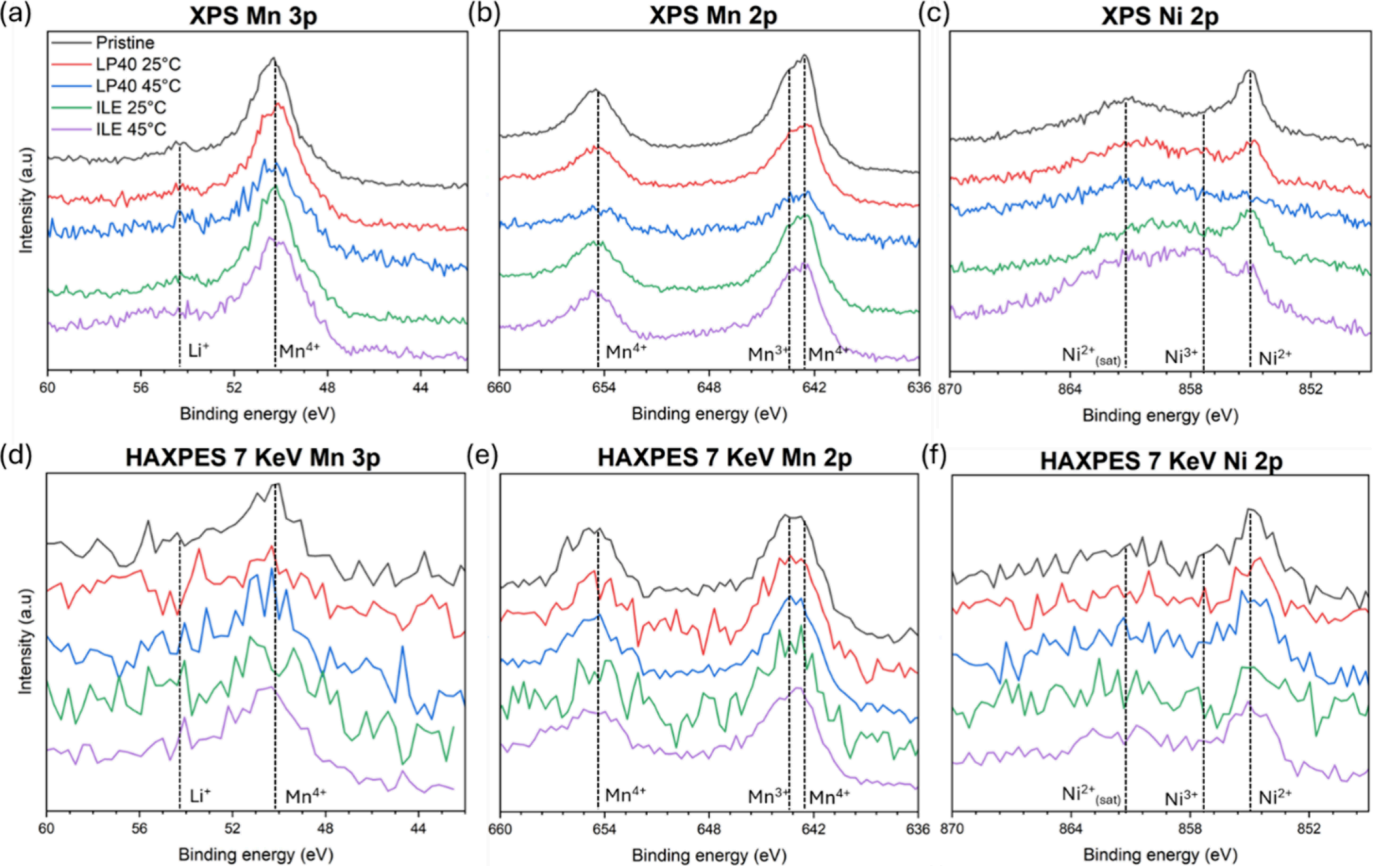
(a–c) XPS and (d–f) 7 keV of Mn 3p, Mn 2p, and Ni 2p of the pristine LNMO cathode and LNMO cathode cycled for 200 cycles in LP40 or ILE at 25 and 45 °C.

Even though the HAXPES signal loses resolution due to the broadening of the signal, the results recorded at 7 keV give an acceptable overview of the state of the LNMO particle at ∼20 nm deep within the sample^61^ (Figure 8d–f). The shapes of all the signals are similar to that of the Pristine material, indicating that the structure of the LNMO deep within the particle is not strongly affected by the cycling and the temperature, which confirms the results observed by HAADF-STEM (Figure 3). When comparing XPS and HAXPES results for Mn and Ni, it appears that LNMO is mostly damaged at its surface.

## 4 Conclusions

Post-mortem STEM analysis showed that the upper ∼22 nm of the LNMO particles cycled in LP40 were damaged, with the deterioration caused by the TMD being mostly made of pits extending around 15–20 nm away from the surface. On the other hand, the structure of the LNMO cycled in ILE was almost identical to that of the pristine material, with the only change being related to a slight increase in the thickness of the (Mn_*x*_Ni_*y*_)_3_O_4_-phase at the surface of the particle, also observed for the pristine electrode (from around 1.5 nm to around 2 nm). In addition, a rock-salt layer of the order of a unit cell was observed on the outermost surface of the electrode cycled in ILE, which was not present in the pristine electrode. From EELS analysis, gradients in Mn, Ni, and O near the surface were obtained, showing an enrichment in Mn and a depletion of O in the upper 22 nm of the surface of the LP40 cycled LNMO, consistent with the formation of a (Mn_*x*_Ni_*y*_)_3_O_4_ on the internal surface of the pits. The Ni profile was relatively constant. XPS analysis showed an abundance of oxygenated carbon bonds at the electrode surface when using LP40 as an electrolyte, while the CEI formed in ILE is thinner.

Overall, the study provides a direct observation of the effects of the TMD when using LP40 electrolyte, while demonstrating that the structural integrity of the LNMO remains intact when the ionic liquid-based electrolyte is employed. Further study on full cells using this ILE would provide valuable insight into the potential upscaling of this system and will be the focus of future research.

## Supplementary Material



## Notes

Cyclic voltammetry of carbon-coated Al foil in LP40 and ILE, SEM and cross section of the LNMO particles, FFTs from some of the STEM images, APT analysis and reconstruction, and deconvoluted XPS of OCV and 45 °C samples (PDF)
