# Supplementary material for: Degradation of LiNi0.5Mn1.5O4 Cathodes in the P111i4FSI Ionic Liquid Electrolyte and Carbonate Electrolytes
Source: ACS Appl Mater Interfaces. 2025 Sep 8;17(37):52112–24. doi: 10.1021/acsami.5c11439 (PMC12447383; doi:10.1021/acsami.5c11439)
Supplement: Supplementary file 1 [file am5c11439_si_001.pdf]

Supporting Information

On the degradation of $LiNi_{0.5}Mn_{1.5}O_4$ cathodes in $P_{111i4}FSI$ ionic liquid electrolyte and carbonate electrolyte.

Johan Hamonnet[a‡*], Inger-Emma Nylund[a‡], Paraskevas Kontis[a], Weicheng Hua[a], Pedro Alonso-Sánchez[b], Juan Rubio Zuazo[c], Maria Valeria Blanco[a], Ann Mari Svensson[a*]

[a]Department of Materials Science and Engineering, Norwegian University of Science and Technology, NO-7491 Trondheim, Norway

[b]Aragon Nanoscience and Materials Institute (CSIC-University of Zaragoza) and Dept. Condensed Matter Physics, Facultad de Ciencias, 50009 Zaragoza, Spain

[c]The spanish CRG beamline, European Synchrotron Radiation Facility, 38000 Grenoble, France

[‡]The authors contributed equally to this work

*Corresponding authors emails: johan.hamonnet@ntnu.no, annmari.svensson@ntnu.no

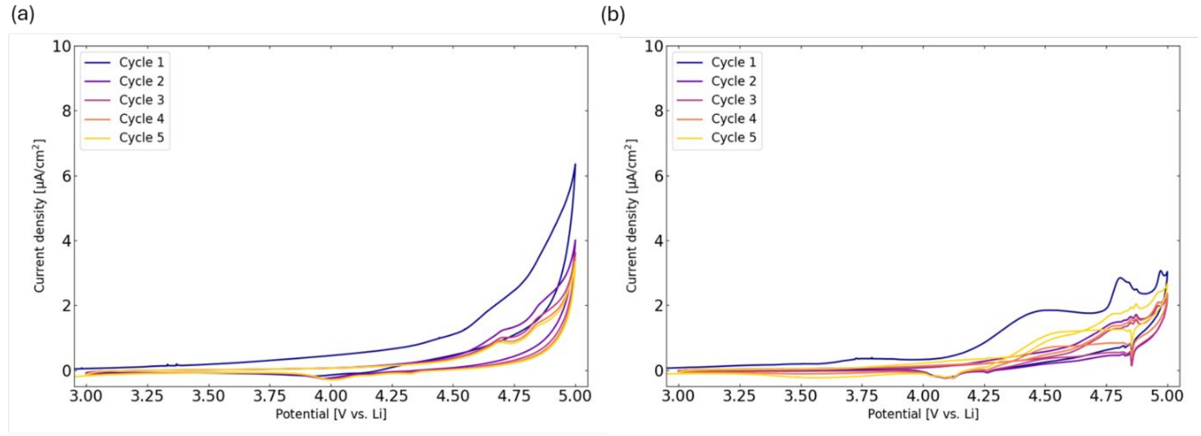

Figure S1: CV of carbon coated Al current collector. (a) In LP40 electrolyte and (b) in LiFSI:P_{111i4}FSI electrolyte.

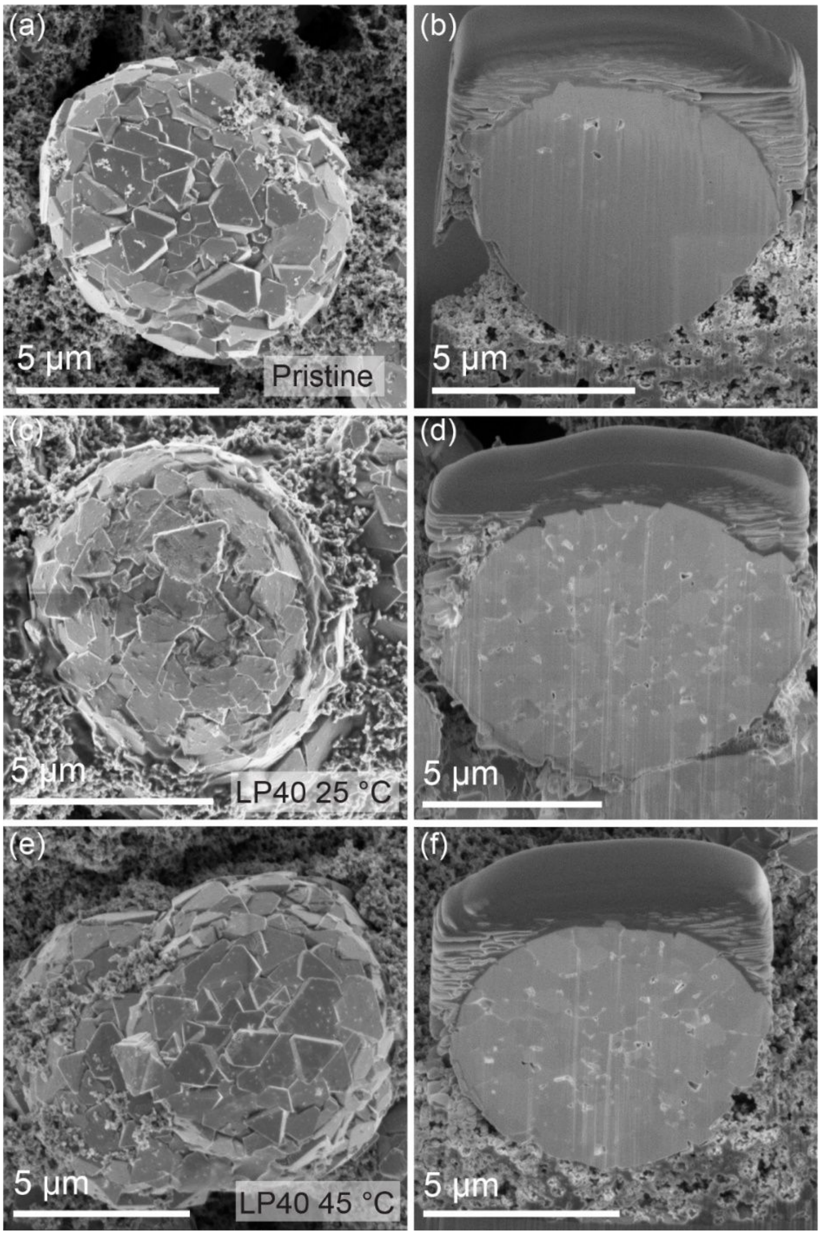

Figure S2. SEM images of a,c,e LNMO particle surfaces and b,d,f LNMO particle cross sections made by FIB. a,b pristine LNMO, c,d after 210 cycles in LP40 at 25 °C, e,f after 210 cycles in LP40 at 45 °C.

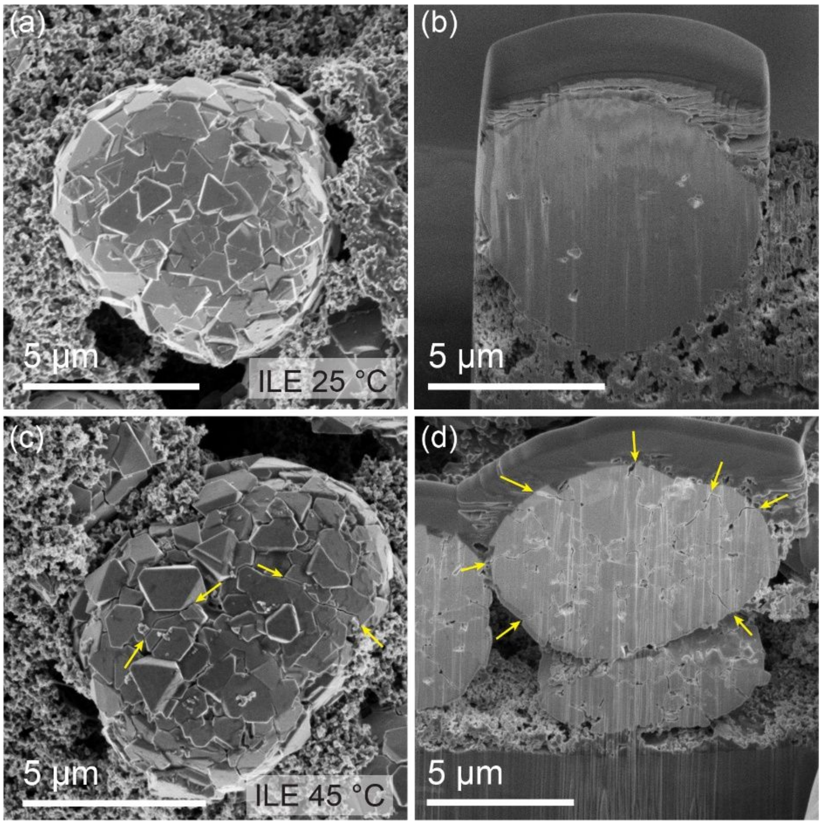

Figure S3. SEM images of a,c LNMO particle surfaces and b,d LNMO particle cross sections made by FIB. a,b after 210 cycles in ILE at 25 °C, and c,d after 210 cycles in ILE at 45 °C. The yellow arrows in c,d indicate the presence of cracks.

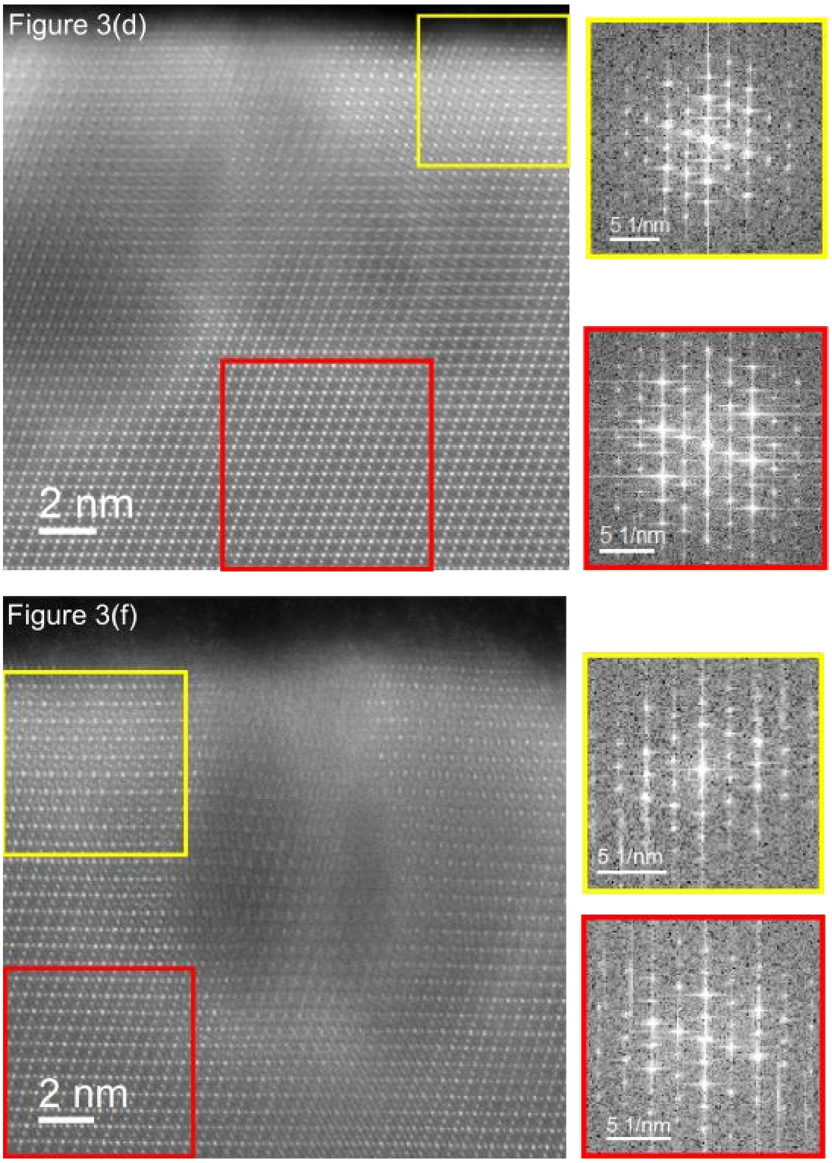

Figure S4. FFTs from two areas of Figure 3(d) and (f) in the main text, indicating a high degree of crystallinity by showing a dense spot pattern.

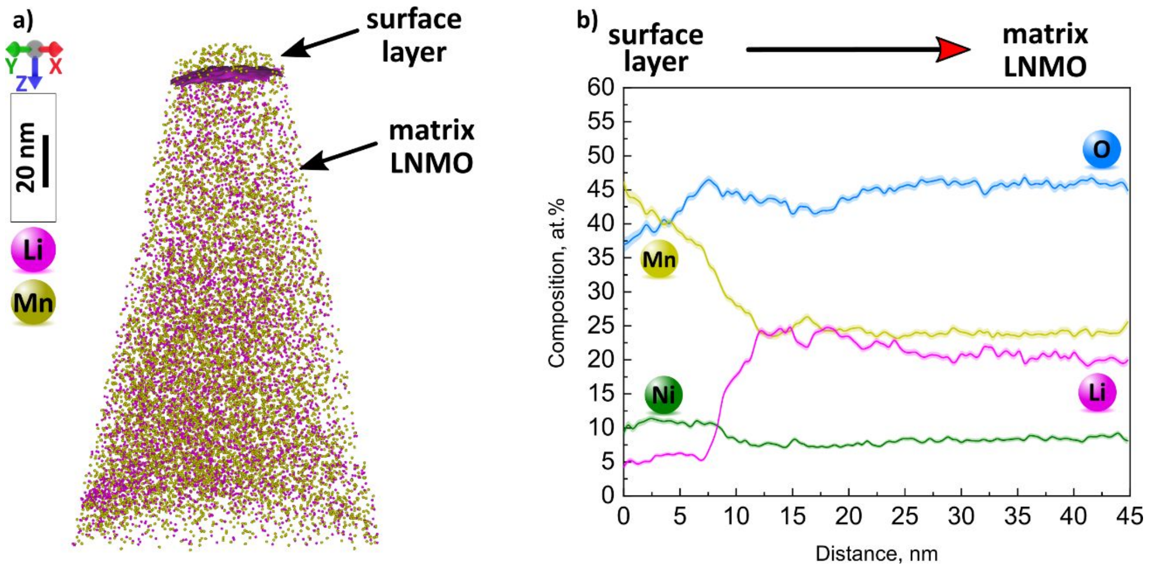

Figure S5. a) An APT reconstruction of the LNMO material cycled 40 times in LP40 at 25 °C. b) 1D concentration profiles from a region of interest perpendicular to the surface layer/matrix interface for Mn, Ni, O, and Li. The Mn and Ni concentrations at the surface increase while the O and Li concentrations decrease. Error bars are shown as lines filled with colour and correspond to the 2σ counting error.

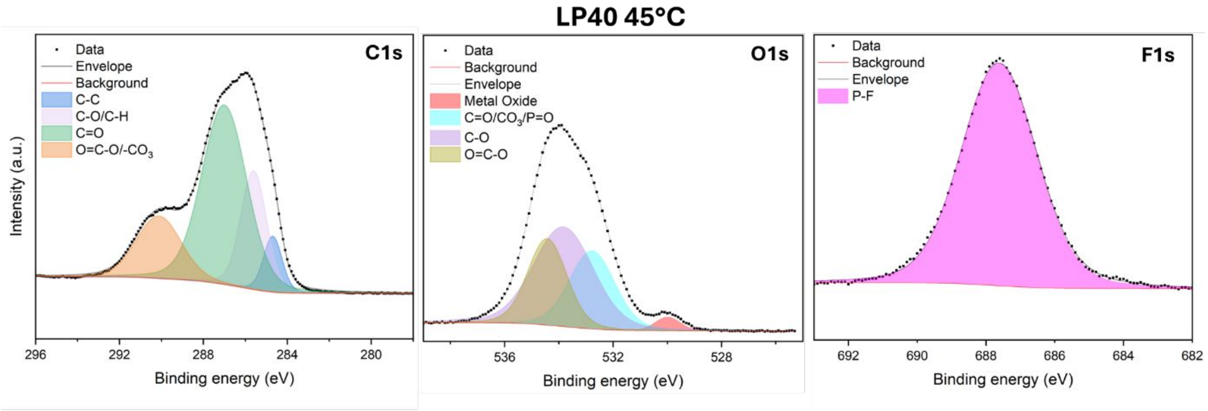

Figure S6. Deconvoluted XPS spectra of C1s, O1s, F1s, P2p, S2p and N1s LNMO cathode cycled for 200 cycles in LP40 at 45°C.

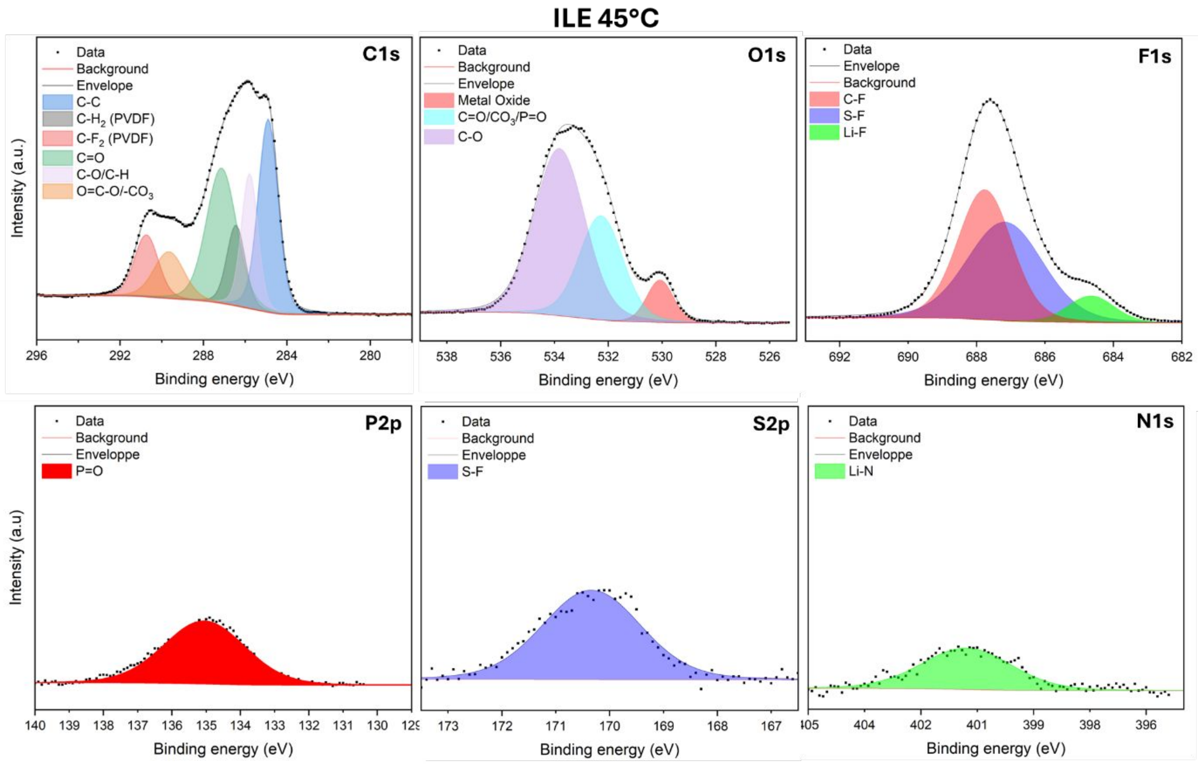

Figure S7. Deconvoluted XPS spectra of C1s, O1s, F1s, P2p, S2p and N1s LNMO cathode cycled for 200 cycles in ILE at 45°C.

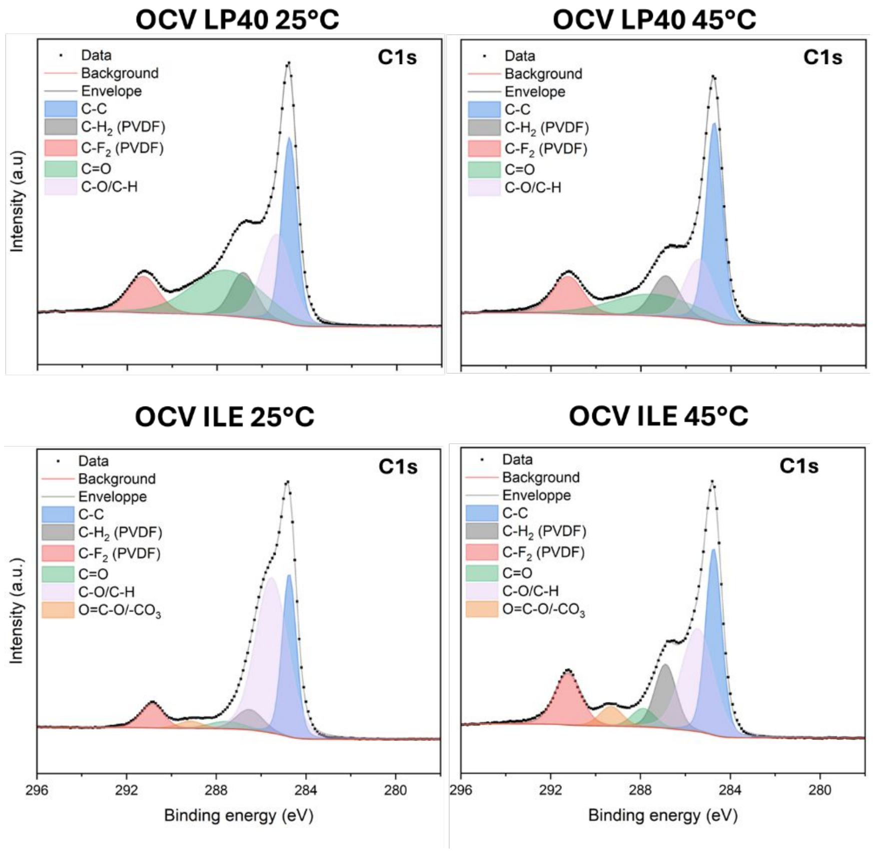

Figure S8. Deconvoluted XPS spectra of C1s for cells kept at open circuit voltage for 1 month with LP40 and ILE electrolytes at 25 and 45 °C

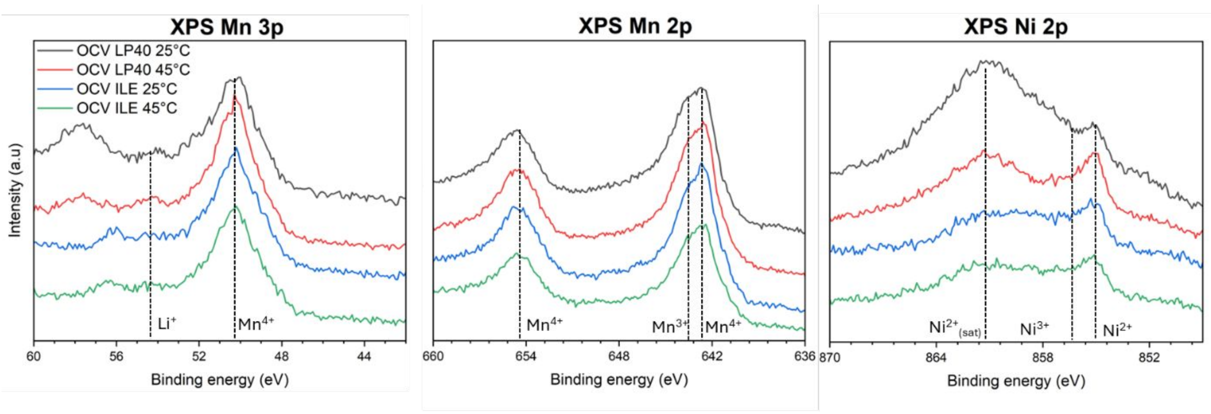

Figure S9. XPS spectra of Mn3p, Mn2p and Ni2p for cells kept at open circuit voltage for 1 month with LP40 and ILE electrolytes at 25 and 45 °C

References:

(1) Devaraj, A.; Gu, M.; Colby, R.; Yan, P.; Wang, C. M.; Zheng, J. M.; Xiao, J.; Genc, A.; Zhang, J. G.; Belharouak, I.; et al. Visualizing nanoscale 3D compositional fluctuation of lithium in advanced lithium-ion battery cathodes. *Nature Communications* **2015**, *6* (1), 8014. DOI: 10.1038/ncomms9014.
